# Giant Trichobezoar Extending to the Ileocecal Junction in a 3-Year-Old Female: A Rare Case Report

**DOI:** 10.7759/cureus.86414

**Published:** 2025-06-20

**Authors:** Sachin Kumar, Rakesh K Tripathi, Naveen K Patel, Kumari Shrestha, Ankush Chauhan

**Affiliations:** 1 General Surgery, Ganesh Shankar Vidyarthi Memorial (GSVM) Medical College, Kanpur, Kanpur, IND; 2 Pediatrics, Government Medical College, Bettiah, Bettiah, IND

**Keywords:** diagnostic imaging limitations, foreign body ingestion, pediatric surgery, rapunzel syndrome, socio-environmental risk

## Abstract

This report details a rare case of a 3-year-old female from a low-income laborer family presenting with a giant trichobezoar extending to the ileocecal junction (ICJ), the youngest documented case with mixed composition (hair, cotton threads, and synthetic materials). Contrast-enhanced computed tomography (CECT) localized the mass to the jejunum, but intraoperative exploration revealed an approximately 145-cm bezoar reaching the ICJ. The case highlights socio-environmental risk factors, diagnostic imaging limitations, and emphasizes the importance of complete surgical exploration in pediatric trichobezoar management.

## Introduction

Trichobezoars, concretions of ingested hair, are rare in children under 5 years and classically linked to trichotillomania [1]. Rapunzel syndrome, where the bezoar extends into the small bowel, is rarer still, with ileocecal junction (ICJ) involvement reported in <15% of cases [2]. Mixed-composition bezoars (e.g., hair with foreign materials) are scarcely documented, often tied to environmental exposure [3]. We present the youngest case of a trichobezoar with ICJ extension in a child of laborers, emphasizing socio-environmental determinants, diagnostic challenges, surgical management, and preventive strategies.

## Case presentation

A 3-year-old female, born to daily-wage textile laborers, presented with a 3-month history of intermittent abdominal pain and non-bilious vomiting. The parents reported no behavioral abnormalities but noted the child’s unsupervised access to discarded clothing materials (threads, sequins). Physical examination revealed a palpable epigastric mass.

**Figure 1:**
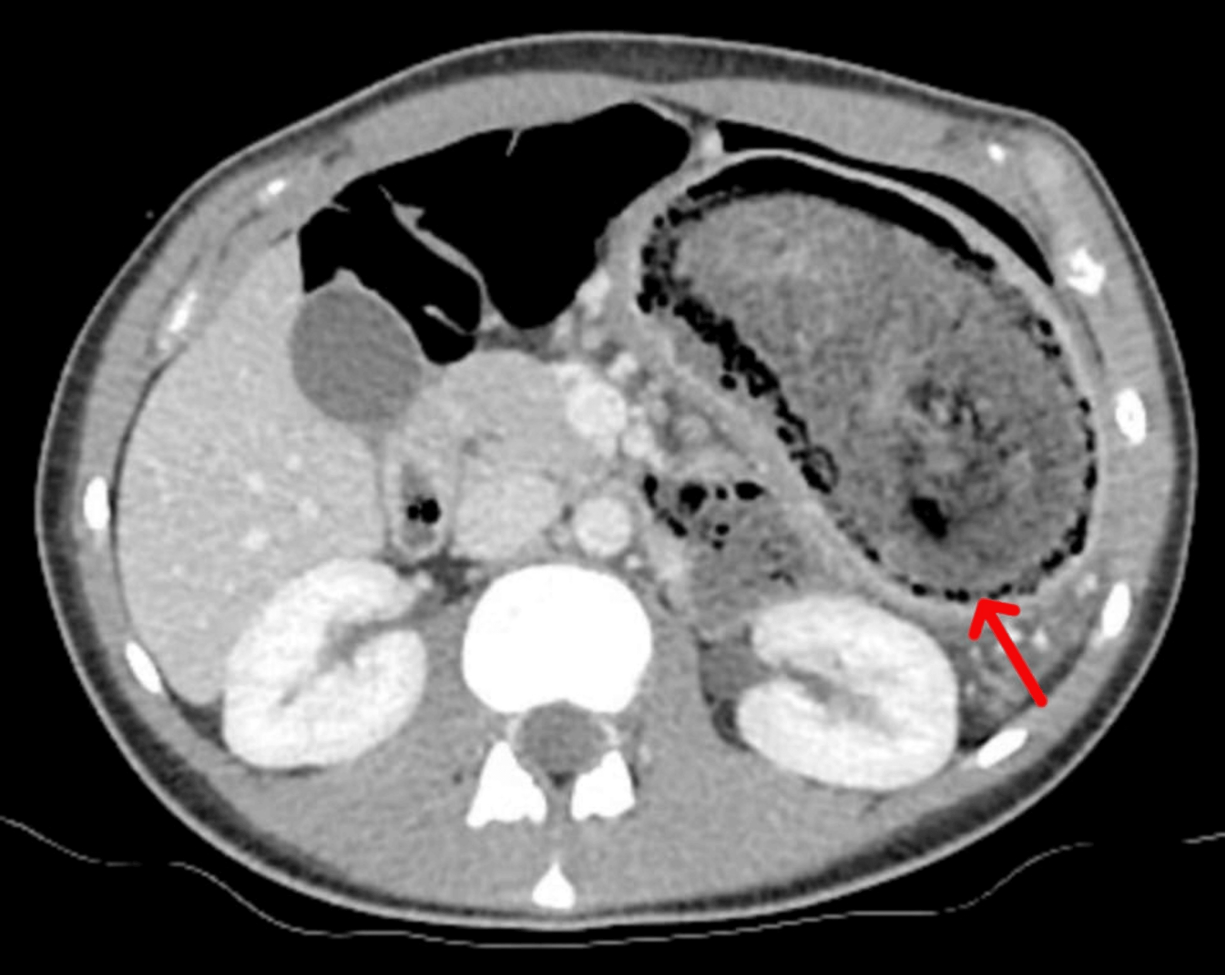
CECT whole abdomen (Axial section) showing heterogeneous intraluminal mass in the stomach, with a mottled gas pattern (Red arrow) CECT: Contrast-enhanced computed tomography

**Figure 2:**
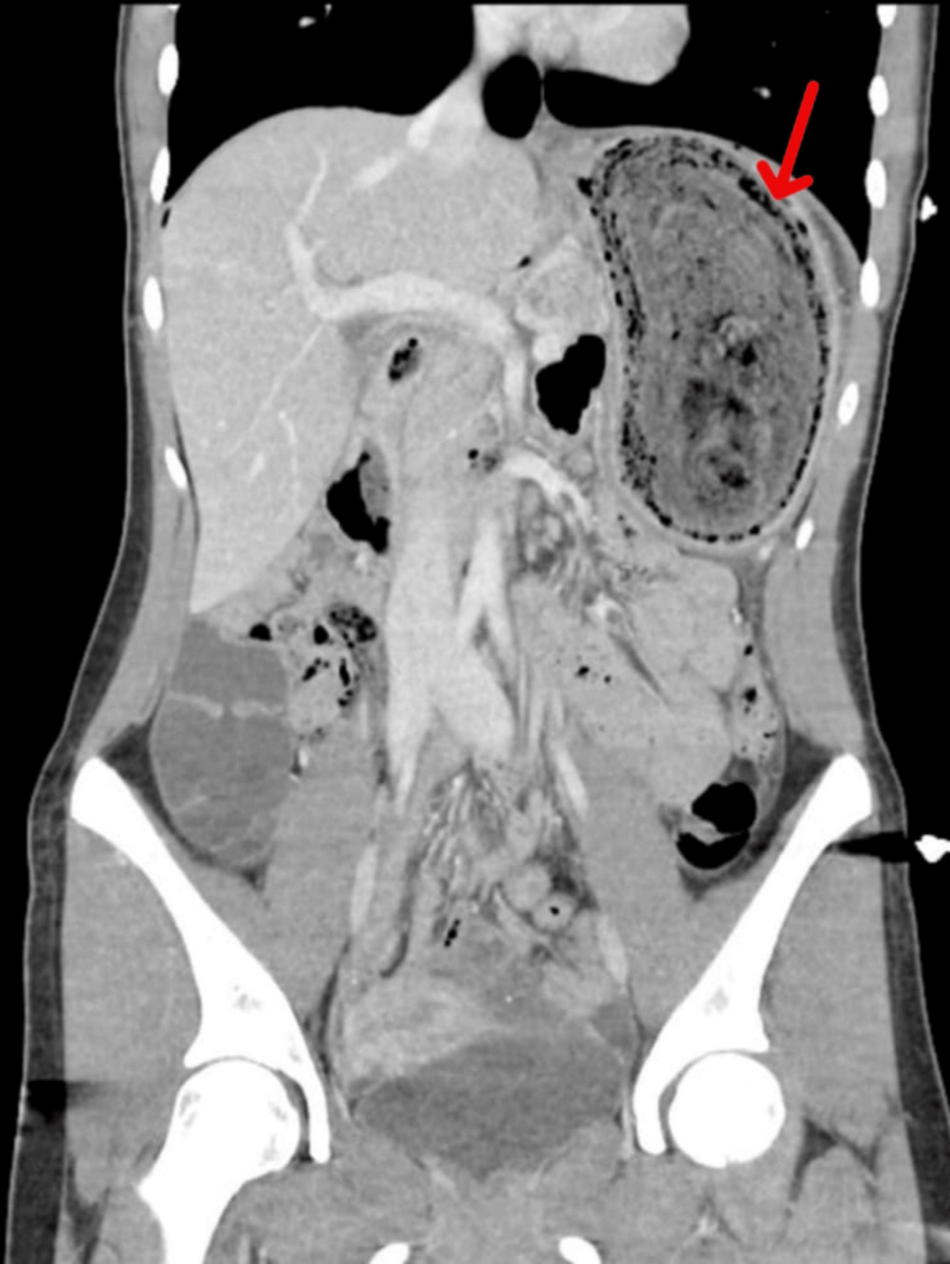
CECT whole abdomen (coronal section) showing heterogeneous intraluminal mass in the stomach extending to the proximal jejunum, with a mottled gas pattern (Red arrow). CECT: Contrast-enhanced computed tomography

Contrast-enhanced computed tomography (CECT) abdomen demonstrated a heterogeneous intraluminal gastric mass (18 × 6 cm) extending into the proximal jejunum with characteristic “mottled gas” appearance (Figures 1-2).

**Figure 3:**
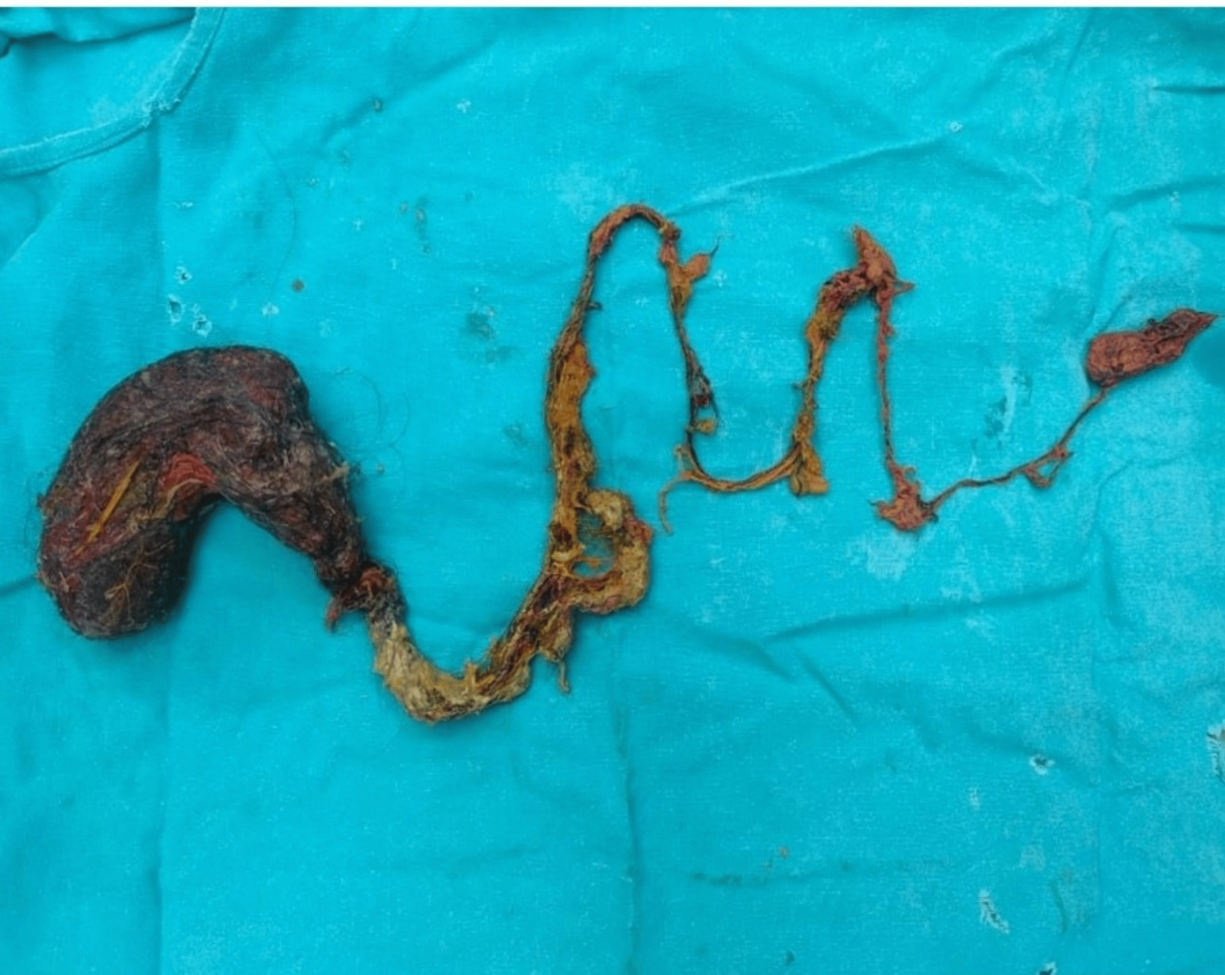
Specimen of trichophytobezoar composed of hair, cotton threads, and synthetic sequins.

Intraoperative exploration revealed an approximately 145-cm trichophytobezoar extending from the stomach through the jejunum and ileum to the ICJ, containing hair, cotton threads, and synthetic sequins (Figure 3). Histopathological examination confirmed the mixed composition.

The bezoar was completely retrieved via exploratory laparotomy and gastrostomy. The patient recovered uneventfully and was discharged on postoperative day 14. Parental counseling for child-proofing the home environment and monitoring for pica, along with referral to pediatric psychiatry for longitudinal behavioral assessment, was done.

## Discussion

The trichobezoar in this case exhibited a unique mixed composition, incorporating not only hair but also cotton threads and synthetic materials commonly found in textile industries. This unusual combination strongly reflects environmental exposure risks that were amplified by the child's parental occupation as textile laborers. Similar cases reported in the literature, such as Kumar et al.'s (2019) description of a 5-year-old with a hair-plastic bezoar, further highlight the particular ingestion risks present in laborer households [3]. The extraordinary length of approximately 145 cm provides compelling evidence of chronic, undetected ingestion over an extended period, emphasizing both the insidious nature of this condition and the challenges in early detection. This finding underscores the need for heightened awareness of environmental risk factors in pediatric populations, particularly those from occupational backgrounds with increased access to potentially ingestible materials.

The imaging-surgical discordance in this case revealed important limitations of current diagnostic protocols. While CECT successfully identified the trichobezoar extending to the jejunum, it failed to detect its full distal extension to the ICJ due to collapsed ileal loops. Although CECT remains the gold standard for initial diagnosis, this case demonstrates its limitations in assessing complete bezoar extent, particularly due to technical challenges like collapsed bowel segments and fecal interference [4]. These findings support the need for improved imaging protocols, such as prone-position scanning, extended oral contrast administration, or magnetic resonance enterography (MRE) to enhance visualization of distal small bowel structures and improve preoperative planning accuracy.

Surgical management of extensive trichobezoars necessitates complete retrieval through thorough intraoperative exploration, as imaging often underestimates the true extent. Our findings corroborate Fallon et al.’s (2013) [2] recommendations for comprehensive surgical assessment regardless of preoperative imaging results, ensuring complete removal and minimizing recurrence risk. This approach proved critical in our case, where the bezoar extended significantly beyond radiological findings.

The child’s background in a low-income laborer family with unrestricted access to textile waste highlights a novel pathway for foreign body ingestion. Parental occupations in industries involving small, ingestible materials (e.g., textiles, plastics) necessitate proactive preventive strategies, including workplace safety education and home environment modifications. This aligns with emerging evidence linking low socioeconomic status to higher rates of accidental ingestion in young children [5]. Community health initiatives targeting high-risk families could mitigate such risks through subsidized childcare or material safety workshops.

Despite the absence of overt psychiatric symptoms, longitudinal behavioral monitoring is essential. Studies indicate that 30-40% of pediatric bezoar cases later develop trichophagia or pica, often linked to undiagnosed anxiety or developmental disorders [6]. Early referral to pediatric psychiatry can preempt recurrence.

At just 3 years old, this case represents the youngest reported instance of a mixed-composition trichophytobezoar extending to the ICJ, significantly expanding the known age range for this condition.

This case challenges conventional assumptions by demonstrating that trichobezoars can occur in very young children without psychiatric comorbidities. The findings compel clinicians to include bezoars in the differential diagnosis of chronic abdominal symptoms even in preschoolers lacking typical hair-pulling behaviors. Furthermore, the mixed composition (hair, textiles, and synthetic materials) highlights the critical importance of histopathological examination of retrieved specimens, as identifying specific ingested materials enables targeted preventive strategies for at-risk populations. These insights expand our understanding of bezoar pathogenesis beyond classic psychiatric associations to include significant environmental determinants.

## Conclusions

This case highlights several critical insights for managing trichobezoars, as imaging modalities like CECT may underestimate the true extent of trichobezoars, emphasizing the need for complete surgical exploration regardless of radiological findings. The mixed composition (hair and textile materials) underscores how environmental exposures in high-risk households can contribute to atypical bezoar formation. While the surgical approach remains standardized, this case reinforces that trichobezoars should be considered even in very young children without classic psychiatric symptoms. Most importantly, it demonstrates that successful management requires both complete physical removal of the bezoar and long-term multidisciplinary follow-up to address potential behavioral components and prevent recurrence. These insights refine our approach to diagnosing and managing this rare but clinically significant condition.
